# Is There a Role for Urodynamic Investigation in the Management of Pelvic Organ Prolapse?

**DOI:** 10.3390/jcm14041163

**Published:** 2025-02-11

**Authors:** Eleonora Rosato, Lorenzo Vacca, Andrea Lombisani, Giuseppe Campagna, Luca Orecchia, Daniele Bianchi, Yuri Cavaleri, Maurizio Serati, Enrico Finazzi Agrò

**Affiliations:** 1 Urology Unit, Department of Surgical Sciences, Policlinico Tor Vergata University Hospital, 00133 Rome, Italy; eleonoraros92@gmail.com (E.R.); luca.orecchia@hotmail.com (L.O.); 2 Gynecological Surgery Unit, Dipartimento Centro di Eccellenza Donna e Bambino Nascente, Ospedale Isola Tiberina, Gemelli Isola, 00153 Rome, Italy; lorenzovacca08@gmail.com (L.V.); andrealombisani1991@gmail.com (A.L.);; 3 Faculty of Medicine and Surgery, University of Rome Tor Vergata, 00133 Rome, Italy; danielebianchimail@yahoo.it (D.B.);; 4 Department of Obstetrics and Gynecology, University of Insubria, 21100 Varese, Italy; mauserati@hotmail.com

**Keywords:** pelvic organ prolapse, urodynamic study, lower urinary tract

## Abstract

**Background/Objectives**: The role of urodynamic study (UDS) in women with pelvic organ prolapse (POP) and concurrent lower urinary tract symptoms (LUTS) remains controversial. Although LUTS alone often fail to yield an accurate diagnosis, routine UDS is debated due to its invasiveness, cost, patient discomfort, and risk of urinary tract infections. The aim of this narrative review is to summarise the utility of UDS in the pre- and postoperative management of POP, focusing on its role in diagnosing and predicting outcomes for detrusor overactivity (DO), bladder outlet obstruction (BOO), detrusor underactivity (DU), and SUI. **Methods**: An extensive search of the available medical literature was conducted using PubMed, Scopus, and Embase to identify relevant studies published up to December 2024. The search combined keywords and MeSH terms related to pelvic organ prolapse (POP), urodynamic studies (UDS), overactive bladder, detrusor overactivity, stress urinary incontinence (SUI), female bladder outlet obstruction (BOO), detrusor underactivity (DU), preoperative assessment, and postoperative outcomes. **Results**: Occult stress urinary incontinence (SUI) detection with UDS can aid in planning concurrent anti-incontinence procedures, while preoperative assessment of DO or DU helps predict postoperative complications like urinary retention or overactive bladder symptoms. **Conclusions**: Despite its diagnostic advantages, evidence on UDS parameters and surgical outcomes remains inconsistent. The most important societies’ guidelines promote the use of UDS in selected cases, highlighting the need for individualised assessments to optimise patient counselling and management strategies.

## 1. Introduction

The role of urodynamic study (UDS) in women presenting with pelvic organ prolapse (POP) and concurrent lower urinary symptoms (LUTS) is unclear. Even though there appears to be a poor correlation between lower urinary tract symptoms (LUTS) and urodynamic findings [1,2], LUTS alone rarely allow physicians to reach the correct diagnosis, sometimes leading to incorrect treatment [3,4]. Current literature has raised concerns about the routinary use of UDS, which should be weighed against the risks/costs, including the time, the invasiveness, the patients’ experience of discomfort, and the risk of post-procedural urinary tract infections (UTIs). The guidelines of most important national and international societies do not recommend performing routinely urodynamics as part of the diagnostic work-up of POP, except in combination with symptomatic stress urinary incontinence (SUI) [5]. During UDS, the use of techniques to reduce POP (Sim’s speculum, gauze pad, and vaginal pessary) to diagnose ‘occult SUI’ is common practice [6]. The detection rate of occult SUI in women without symptoms increased (4.4% vs. 22%) in cases of reduction with a pessary independently of POP stage [7]. In particular, the presence of SUI may be used to decide if additional anti-UI surgery should be offered at the time of POP repair or to counsel patients on the possible SUI-related symptoms after POP treatment.

Furthermore, information about detrusor activity deriving from UDS may provide information about the risk of persistent or de novo detrusor overactivity (DO) after surgery, as well as the risk of urinary retention (UR) due to detrusor impairment [8]. Preoperative maximum urethral closure pressure (MUCP), peak flow rate, and maximum detrusor voiding pressure are independent risk factors for postoperative DO [9]. Instead, a rate of 47% of women who underwent POP repair normalised urodynamic findings after surgery in cases of detrusor underactivity (DU) diagnosis [10].

The aim of this narrative review is to summarise the evidence on the role of UDS in women with POP, emphasising how its routinely use permit the personalisation of treatment strategies. Furthermore, it highlights the potential benefits of incorporating UDS into routine pre- and postoperative care to improve counselling and optimise management.

## 2. Materials and Methods

A comprehensive search of the medical literature was performed using the PubMed, Scopus, and Embase databases to identify relevant articles published up to December 2024. The search strategy included a combination of keywords and MeSH terms such as ‘pelvic organ prolapse’, ‘urodynamic studies’, ‘overactive bladder’, ‘detrusor overactivity’, ‘stress urinary incontinence’, ‘female bladder outlet obstruction’, ‘detrusor underactivity’, ‘preoperative assessment’, and ‘postoperative outcomes’.

Included studies were original articles (randomised controlled trials, case series, cohort studies, case-control studies, etc.) that addressed the use of UDS in the context of POP, focusing on its diagnostic utility, prognostic value, and impact on patient management and surgical outcomes and the guidelines of most important urogynaecological societies. We excluded articles that were not published in English (e.g., in Japanese or Chinese language) to minimise the risk of misinterpretation. To ensure that important findings were not overlooked, we reviewed the corresponding abstracts. Additionally, editorials, case reports, and letters to the editor were excluded from the analysis.

The selection process was carried out in two stages: first, titles and abstracts were screened for relevance by two authors (A.L. and L.V.), and then full texts of potentially eligible articles were reviewed by another author (E.R.). Reference lists of selected studies were also screened to identify additional relevant publications.

To balance the intrinsic risk of bias and heterogeneity of the included studies, the findings were summarised and discussed in the context of current clinical practice and guidelines, with an emphasis on identifying gaps in knowledge and areas for future research.

## 3. Results

We identified 150 articles based on our search strategy. After the initial screening, 52 articles were included in this narrative review. To summarise the findings, we reported the results based on correlations between POP and preoperative urodynamic parameters. Additionally, we examined the correlation between UDS and postoperative outcomes following POP repair.

### 3.1. Role of UDS in POP with Overactive Bladder (OAB) and Detrusor Overactivity (DO)

Overactive bladder (OAB) symptoms are more common in patients with POP (risk ratio, RR = 5.8), with a prevalence of up to 57% [11]. Furthermore, observational studies report a higher prevalence of DO in POP (10–50%), though UDS use as the gold standard is debated. Theories suggest that POP may cause OAB and DO by inducing bladder changes, such as neurological damage or structural alterations to the detrusor muscle and its receptors [12]. If POP may be considered a risk factor for BOO and OAB or DO, it can be hypothesised that resolving BOO through POP repair could lead to improvements in OAB symptoms, DO, and urge urinary incontinence (UUI) [8,13].

The presence of pre-existing and the emergence of de novo OAB represent a substantial challenge in the context of prolapse surgery. Johnson et al. reported that 61% of patients experienced resolution of OAB symptoms after POP surgery, regardless of incontinence procedure inclusion, with older age linked to persistent symptoms [14]. In a Cochrane review, 12% of patients developed de novo OAB symptoms after POP surgery, independently of the surgical approach [15] and if a concomitant anti-incontinence procedure was performed [15]. Age, sling placement, presence of postoperative SUI or voiding symptoms, and postoperative serious constipation or preoperative rectocele were found to be independently associated with de novo OAB [16,17].

It is important to emphasise that the studies have not established a correlation between UDS and the presence of OAB symptoms and DO, either pre- or postoperatively. However, OAB symptoms and DO significantly impact the quality of life (QoL) in patients with POP. Several authors have developed predictive models to identify patients at the highest risk of developing de novo or persistent OAB and DO after surgery. Although UDS plays a relevant role in context, it has yet to become a standard evaluation in risk assessment.

Preoperative DO is linked to an increased risk of OAB symptoms and their persistence post-surgery [18,19,20,21]. Other preoperative UDS parameters such as maximum bladder capacity (MCC) < 300 mL, severe bladder trabeculation, and a duration of POP symptoms > 60 months, were associated with persistent UUI following prolapse repair [22]. In high-grade POP patients, predictors of persistent urgency include a lower maximum flow rate on UDS (13.9 mL/s vs. 15 mL/s, *p* = 0.04), preoperative DO (OR: 12.2 [95% CI: 1.4–16.6], *p* = 0.01), UUI (OR: 3.8 [95% CI: 1.3–11.0], *p* = 0.008), and BMI >25 kg/m^2^ (OR: 1.8 [95% CI: 1.1–7.2], *p* = 0.04) as significant predictors [23]. A large retrospective study identified maximum urethral closure pressure (MUCP) ≥ 60 cmH_2_O, maximum flow rate (Qmax) < 15 mL, detrusor pressure at maximum flow rate (pdetQmax) ≥ 20 cmH_2_O, and post-void residual (PVR) ≥ 200 mL, along with older age and neurological disease, as independent risk factors for postoperative DO after vaginal POP surgery [9].

The findings from the included studies were summarised and discussed in the context of current clinical practice and guidelines, with an emphasis on identifying gaps in knowledge and areas for future research.

### 3.2. Role of UDS in POP with Stress Urinary Incontinence (SUI)

Approximately 50% of patients with POP report stress urinary incontinence (SUI) prior to surgery. Of these, 35% experience resolution, 14% show improvement, and 5.1% require additional procedures for persistent SUI following POP surgery [24]. The findings from the few studies investigating the outcomes of POP surgery in patients suffering from SUI have been inconsistent. The considerable heterogeneity in the results do not permit a discernible correlation between UDS parameters and the resolution of SUI after surgery.

However, the presence of SUI on preoperative UDS has been hypothesised as a risk factor for persistent SUI following surgery (odds ratio, OR 3.11) [25,26]. Lower preoperative maximum urethral closure pressure (MUCP; *p* = 0.031) and higher body mass index (BMI) have been identified as risk factors [27]. A retrospective study indicated that clinically incontinent patients with MUCP ≤ 50 cmH_2_O derive the greatest benefit from concomitant POP and SUI surgery [28]. In the Illiano et al. series, SUI disappeared in 65% of women after sacrocolpopexy without any concomitant anti-incontinence surgery [29]. Abdullah et al. found an incidence of SUI at 42.2%, of which 26.6% was de novo SUI [27].

#### 3.2.1. POP and De Novo SUI

De novo SUI may develop in 15% of cases after POP surgery when concurrent incontinence procedures are not performed, regardless of preoperative symptoms. The risk is significantly higher (59–65%) in patients with positive occult SUI testing or clinical symptoms before surgery. In a study by van der Ploeg et al., only 9% of women without SUI who skipped anti-incontinence procedures during POP repair developed de novo SUI, and 3.5% required surgery [30]. Notably, all women treated for de novo SUI had shown signs of SUI during the initial office evaluation [30]. Additionally, over 50% of patients with high grade POP show SUI on UDS and 40% of them are asymptomatic, highlighting the need for thorough preoperative urodynamic evaluation [31].

Some authors have created models to predict de novo SUI by combining patient characteristics and urodynamic parameters. However, computer-based models using preoperative symptoms and baseline characteristics failed to replace preoperative UDS [1]. Following transvaginal pelvic reconstructive surgery, some general aspects and UDS parameters were independent risk factors for de novo SUI, such as advanced age (>66 years), functional urethral length <2 cm, MUCP <60 cmH_2_O, SUI diagnosed via UDS (OR 2.26), and detrusor pressure at maximum flow (Pdet at Qmax) < 30 cmH_2_O (OR 2.93) [25,32]. In another study, lower maximum urethral closure pressure (MUCP) (<40 cmH_2_O) was linked to higher de novo SUI risk after sacrocolpopexy [33].

These findings corroborate the ambiguous role of urodynamic testing in the preoperative assessment of SUI recovery and persistent or de novo SUI after prolapse repair [32].

#### 3.2.2. POP and Occult SUI

Most of the clinical research in the field of urodynamics in patients with POP has been focused on patients with ‘occult SUI’. The objective of identifying ‘occult SUI’ in patients with POP is to administer specific treatment, often combined with anti-incontinence procedures.

In a recent review, preoperative occult SUI diagnosed by preoperative UDS has been found to have a positive predictive value (PPV) for de novo SUI of 40% (0–79%), and its absence has been found to have a negative predictive value (NPV) of 91% (51–100%), respectively [18]. A retrospective cohort study revealed that among women with UDS-detected occult SUI, postoperative SUI rates at one year were 8.8% (subjective) and 12.5% (objective) for those undergoing combined POP and anti-incontinence surgery, compared to 18.2% and 42.9% for POP surgery alone. For women with negative UDS results, these rates were 0.0% and 50.0% with combined surgery, and 12.8% and 27.6% with POP surgery alone. No significant differences were found in outcomes based on the procedure for those with positive occult SUI [34].

While UDS is sensitive in diagnosing occult SUI, its practical usefulness is debated. A retrospective study of 392 women with LUTS found that UDS rarely altered surgical plans or counselling (3.5%), except for cases involving SUI with voiding symptoms or elevated PVR [13]. Another study reported that UDS results influenced treatment plans in 44.2% of cases, helping to exclude unfavourable factors in 22.4% [35].

### 3.3. Role of UDS in POP with Bladder Outlet Obstruction (BOO)

POP can cause mechanical obstruction of the urinary system, leading to increased urinary resistance, incomplete bladder emptying, and secondary changes in the detrusor. BOO is often due to urethral compression associated with apical, posterior, or anterior compartment prolapse and is more common in advanced stages [36,37,38]. However, there are no standardised urodynamic thresholds for diagnosing BOO in women [39]. The only validated nomogram for women is the Blaivas–Groutz nomogram, but it does not fully capture all aspects of BOO and detrusor contractility and other urodynamic criteria for evaluating BOO in women were proposed [5]. Few studies evaluate female BOO and demonstrate that half of all women with POP have a feeling of incomplete bladder emptying, but only 30% of the patients with advanced POP have urodynamic evidence of BOO [40]. In a study including 74 patients who underwent UDS before POP repair, 35% patients presented voiding LUTS (hesitancy, abdominal straining, weak interrupted urine stream, and sense of incomplete emptying). Of those, 77% had abnormal voiding cystometry with an obstructed flow and high PVR [19].

BOO due to POP is successfully treated after POP correction or with pessary reduction. Up to 74% of patients who had the cystocele repaired with anterior colporrhaphy or a polypropylene mesh repair had an improvement in their voiding difficulties [41]. Those with improvement had significantly higher preoperative PVR volumes compared to those without improvement. Symptom relief was not associated with cystocele grade but was correlated with older age [41]. In another study, Romanzi and colleagues evaluated 60 women with grades 1–4 POP to assess whether prolapse that causes BOO (defined as a detrusor pressure > 25 cm H_2_O and maximum flow < 15 mL/s) can be relieved by pessary reduction. They found that 70% of women with grade 3–4 POP had urodynamic BOO, compared to only 3% with grade 1–2 POP. After prolapse reduction with a ring pessary, 94% of women with grade 3–4 POP and BOO showed normal free uroflowmetry. Finally, Mueller and colleagues found no significant differences in voiding UDS parameters (MCC, voided volume, pdetQmax) with or without prolapse reduction but a significant decrease in MUCP was observed. They found that 40% of patients met the obstruction using Romanzi et al. criteria [37] without prolapse reduction, compared to only 17% with reduction [42].

According to the American Urological Association (AUA) and the Society of Urodynamics, Female Pelvic Medicine and Urogenital Reconstruction (SUFU) guidelines, urodynamic studies with POP reduction can help assess detrusor dysfunction in women with LUTS [43]. Some researchers suggest considering elevated PVR or DU in protocols for anti-incontinence procedures during POP repair, but simultaneous sling placement is generally avoided because the high-risk patient group is poorly defined [44].

### 3.4. Role of UDS in POP with Detrusor Underactivity (DU)

Diagnosing detrusor underactivity (DU) in women is challenging. Women often void at very low detrusor pressure (Pdet), due to low urethral resistance caused by pelvic floor muscle relaxation or a weak bladder outlet [5]. UDS is crucial for evaluating these patients and confirming the diagnosis of DU because the definitive indicator of this condition is an acontractile detrusor, defined as no detrusor pressure during voiding, with the patient voiding solely by straining [45]. Other suggested parameters include PdetQmax < 30 cmH_2_O, Qmax < 10 mL/s, bladder voiding efficiency (BVE) < 90%, reduced maximum cystometric capacity, and impaired compliance [45].

POP is linked to various lower urinary tract dysfunctions, with its association with DU being the least supported by evidence, partly due to ongoing debates and lack of consensus on DU definitions [46]. The presence of DU may be directly related to the severity of anterior compartment prolapse according to POP-Q evaluation. It suggests that BOO and the chronically increased urethral resistance might be strongly related to the development of DU in POP patients [46].

In 518 women with POP, according to bladder contractility index (BCI), DU was identified in 212 patients (40.9%). These women had higher PVRs, voiding symptoms, and greater anterior compartment descent [46]. In another study including 49 women with POP≥3 with DU (defined as a P_det_Q_max_ of <10 cmH_2_O and a Q_max_ of <12 mL/s), following pelvic reconstructive surgery, 57% recovered bladder contractions, which were absent before surgery [10].

In a Tawfeek et al. study, following POP repair without concomitant incontinence procedures, 15% had persistent sense of incomplete emptying even though postoperative UDS showed a significant reduction of PVR (*p* = 0.0003). All these patients had DU and a mild obstruction both on preoperative and postoperative UDS [19]. In this context, UDS may help to determine if elevated PVR is due to BOO or DU or both. UDS is particularly valuable for patients with voiding symptoms and those scheduled for POP repair, especially if DU is suspected (e.g., in cases of neurological conditions, diabetes, advanced age, or prior pelvic surgery). This approach facilitates optimal counselling regarding the potential persistence of voiding LUTS after POP repair.

## 4. Discussion

The diagnostic role and clinical meaning of urodynamic testing in POP and urinary incontinence (UI) are controversial. Preoperative UDS is usually performed before urogynaecological procedures for urinary incontinence (UI) and pelvic organ prolapse (POP). It represents the only investigational tool able to explain in an objective and reproducible manner the pathophysiology of urogynaecological symptoms and to help physicians in the counselling for patients and in therapeutic choice. It can help better characterise voiding dysfunction and provides supplementary information that may not be achieved from a thorough history and physical examination. However, conducting high-quality RCTs to accurately assess the role of UDS in POP management remains challenging. These difficulties stem from defining an appropriate study population, ensuring an adequate sample size, and determining a sufficient follow-up duration [47]. Moreover, as demonstrated by Tarcan et al., when all patients receive the same treatment regardless of UDS findings—as observed in previous trials—the study fails to evaluate the true impact of UDS on treatment decisions [47]. Inevitably, this limitation in existing studies affects the generalisability of findings reported in the literature. Table 1 provides a summary of the available UDS parameter data and our perspective on how these findings may impact outcomes and patient counselling in high-risk groups.

In patients with POP and concomitant LUTS, the AUA/SUFU recommended the UDS execution to assess occult SUI and detrusor dysfunction once the POP has been surgically repaired (Evidence Strength: Grade C) [43,48,49].

The European Association of Urology (EAU) guidelines recognise that UDS may be valuable to choose the ideal management strategy, and it may reveal occult SUI. However, its predictive value is questioned [20].

In 2017, the recommendations published by French most important societies (Association française d’urologie -AFU, Collège national des gynécologues et obstétriciens français -CNGOF-, Société interdisciplinaire d’urodynamique et de pelvi-périnéologie -SIFUD-PP-, Société nationale française de colo-proctologie -SNFCP-, and Société de chirurgie gynécologique et pelvienne -SCGP-) recommended the execution of UDS assessment in women with POP only in the presence of spontaneous or occult UI (Grade C of recommendation) [50].

The National Institute for Health and Care Excellence (NICE) agrees that a urodynamic exam can be beneficial if the diagnosis is unclear or if the woman has symptoms of voiding dysfunction, anterior or apical prolapse, or a history of surgery for stress urinary incontinence [51].

In 2020, the Italian Society of Urodynamics (SIUD) published a position statement on the diagnostic significance of UDS in patients with POP, UI, and OAB [4]. According to this review, the role of urodynamic investigations in women with POP has not been fully established. While UDS could be useful in the prediction of postoperative bladder function after surgical pelvic floor repair, it does not appear able to significantly influence the surgical treatment. For this reason, SIUD discourages the routinary use of UDS in women with POP, recommending its use only before prolapse surgery, in those cases in which its results may change preoperative planning and/or postoperative prognosis [4].

Finally, in 2016, the working group of the International Continence Society (ICS) Standardisation Steering Committee updated the ICS Good Urodynamic Practice published in 2002 [52]. According to their manuscript, UDS should not be performed without precise indications and well-defined ‘urodynamic questions’ that are to be answered by the outcomes of the urodynamic evaluation [52]. Particularly, UDS evaluation is recommended when the results may change management, such as prior to most invasive treatments for UI and POP [5]. All societies’ recommendations are summarised in Table 2.

In conclusion, while numerous guidelines emphasise the importance of limiting the use of UDS to scenarios where it may influence clinical decision-making, none offer an analysis of its cost-effectiveness in patients with POP. This constitutes a notable gap in the existing literature and underscores the need for further research to assess the relationship between its use and economic implications and the impact of its lack in low resources setting. Another significant limitation of the cited guidelines is their failure to provide a definitive recommendation, even one based on expert panel consensus, regarding the selection of specific patients who should undergo urodynamic testing in the preoperative or postoperative setting. This lack of explicit guidance highlights the necessity for further research to more precisely establish the role of UDS in clinical practice. The integration of artificial intelligence (AI) systems could represent a valuable tool to identify and prioritise the most suitable patients for pre- and postoperative UDS, thereby enhancing its clinical utility and optimising its cost-effectiveness.

## 5. Conclusions

The role of UDS in the evaluation and management of POP remains debated. Although UDS provides objective and reproducible data that may clarify complex pathophysiological conditions, its routine use is not universally recommended by national and international societies. The available data highlight the utility of UDS in patients with concomitant LUTS, stress urinary incontinence, or suspected detrusor impairment. UDS may predict postoperative outcomes and guide surgical decisions when combined with clinical and patient-reported data.

However, the lack of standardised definitions in the female population, combined with conflicting findings in the literature regarding its predictive value, limits its widespread application. Despite these limitations, UDS remains an essential tool in tailored patient management, particularly for those at higher risk of persistence or de novo LUTS after POP repair.

Future research should focus on refining diagnostic criteria, improving predictive models (e.g., using artificial intelligence), and conducting high-quality randomised controlled trials to establish clearer guidelines for its use.

## Figures and Tables

**Table 1:** Summary of the impact of UDS findings on the high-risk patients management.

High-Risk Patient Category	Indications for UDS	UDS Findings	How UDS May Improve Patients Management
Patients with Overactive Bladder (OAB) or Detrusor Overactivity (DO)	Presence of OAB symptoms, urgency urinary incontinence (UUI)	Lower maximum flow rate (13.9 mL/s vs. 15 mL/s, *p* = 0.04), preoperative DO (OR: 12.2 [95% CI: 1.4–16.6], *p* = 0.01) and UUI (OR: 3.8 [95% CI: 1.3–11.0], *p* = 0.008) [23] MUCP ≥ 60 cmH_2_O, maximum flow rate (Qmax) < 15 mL, pdetQmax ≥ 20 cmH_2_O, and post-void residual (PVR) ≥ 200 mL [9] MCC < 300 mL associated with bladder trabeculation and POP > 60 months [22]	UDS can help identify preoperative DO, which is linked to persistent OAB symptoms postoperatively
Patients with Stress Urinary Incontinence (SUI) or Occult SUI	Preoperative SUI symptoms, high-grade POP, or positive occult SUI test	Lower preoperative MUCP ≤ 50 cmH_2_O and higher body mass index (BMI) [27,28]	UDS can predict postoperative SUI persistence and guide decision-making on possible concomitant anti-incontinence procedures
Patients at Risk of De Novo SUI	High-grade POP (Stage III–IV), and functional urethral length < 2 cm	Pdet at Qmax < 30 cmH_2_O (OR 2.93) [25,32]	UDS helps predict and stratify risk for de novo SUI after POP surgery
Patients with Bladder Outlet Obstruction (BOO)	Symptoms of voiding dysfunction and PVR > 200 mL)	Detrusor pressure during voiding phase > 25 cmH_2_O and maximum flow < 15 mL/s) [37]	UDS can differentiate BOO from detrusor underactivity (DU) and guide surgical planning
Patients with Detrusor Underactivity (DU)	History of voiding difficulty, diabetes, or neurological conditions	POP ≥ 3 with PdetQmax < 10 cmH_2_O and Qmax < 12 mL/s [10]	UDS can confirm DU and assist in counseling about potential postoperative voiding dysfunction

**Table 2:** Most important societies’ recommendations on the use of UDS in women with POP.

Society	Recommendation	Grade of Recommendation
SUFU/AUA	To assess occult SUI and DU with POP reduction	Grade C
AFU	To assess occult SUI	Grade C
SIUD	When the results could be useful for diagnosis, preoperative planning, severity assessment, and prognosis of surgical treatment	NA
NICE	When POP is associated with symptomatic SUI	NA
EAU	To detect occult SUI	NA
ICS	When result may change management	NA

## Data Availability

No new data were created or analysed in this study. Data sharing is not applicable to this article.

## Abbreviations

The following abbreviations are used in this manuscript:

UDS	Urodynamic study
POP	Pelvic organ prolapse
LUTS	Lower urinary tract symptoms
SUI	Stress urinary incontinence
DO	Detrusor overactivity
BOO	Bladder outlet obstruction
DU	Detrusor underactivity
UTIs	Urinary tract infections
MUCP	Maximum urethral closure pressure
OAB	Overactive bladder
UUI	Urge urinary incontinence
MCC	Maximum bladder capacity
BMI	Body mass index
PPV	Positive predictive value
NPV	Negative predictive value
AUA	American Urological Association
SUFU	Society of Urodynamics, Female Pelvic Medicine and Urogenital Reconstruction
BVE	Bladder voiding efficiency
BCI	Bladder contractility index
UI	Urinary incontinence
EAU	European Association of Urology
AFU	Association française d’urologie
CNGOF	Collège national des gynécologues et obstétriciens français
SIFUD-PP	Société interdisciplinaire d’urodynamique et de pelvi-périnéologie
SNFCP	Société nationale française de colo-proctologie
SCGP	Société de chirurgie gynécologique et pelvienne
NICE	National Institute for Health and Care Excellence
SIUD	Italian Society of Urodynamics
ICS	International continence society
